# A Two-Stage Framework for Early Detection and Subtype Identification of Alzheimer’s Disease Through Multimodal Biomarker Extraction and Improved GCN

**DOI:** 10.3390/brainsci16030255

**Published:** 2026-02-25

**Authors:** Junshuai Li, Wei Kong, Shuaiqun Wang

**Affiliations:** College of Information Engineering, Shanghai Maritime University, 1550 Haigang Ave., Shanghai 201306, China; lijunshuai@stu.shmtu.edu.cn (J.L.); wangsq@shmtu.edu.cn (S.W.)

**Keywords:** Alzheimer’s disease, mild cognitive impairment, multimodal data integration, machine learning, biomarkers, subtype identification

## Abstract

Background: Imaging-transcriptomic analysis, through the integration of multimodal magnetic resonance imaging (MRI) and transcriptomic data, provides complementary structural, functional, and molecular information that is crucial for the early detection and mechanistic exploration of Alzheimer’s disease (AD). However, effectively extracting features from heterogeneous multimodal data and capturing the associations between microscopic molecular variations and macroscopic brain alterations remain key challenges. Recent advances in deep learning and multimodal integration have enhanced the ability to model nonlinear cross-modal relationships, enabling more accurate identification of imaging-transcriptomic biomarkers and subtypes. Developing robust multimodal frameworks is therefore essential for early AD detection, subtype identification, and advancing precision medicine in neurodegenerative diseases. Methods: In this study, a two-stage method of multimodal Feature Extraction based on Association Analysis and Graph Convolutional Network with Self-Attention and Self-Expression framework (MFEAA-GCNSASE) for early diagnosis of AD and effective identification of subtypes of MCI with different progression to AD is proposed. In the first stage, the MFEAA model is applied to integrate multiple association analysis methods on sMRI, PET, and transcriptomic data to identify key multimodal biomarkers for AD and mild cognitive impairment (MCI). In the second stage, the GCNSASE model enhances classification accuracy between AD and MCI patients through self-attention and self-expression layers. Additionally, unsupervised clustering was performed on MCI samples using top multimodal biomarkers to explore subtype heterogeneity and conversion risk. Reliable MCI subtypes were also identified through a consensus clustering approach. Results: The proposed algorithm integrates sMRI, PET, and transcriptomic data, identifying robust biomarkers including the Left Hippocampus, Left Angular Gyrus, and key genes such as SLC25A5 and GABARAP. To ensure statistical robustness given the extreme class imbalance, we employed a rigorous repeated stratified cross-validation (RSCV) framework. GCNSASE achieved state-of-the-art discrimination performance with mean AUC values ranging from 0.946 to 0.961 across feature subsets (10–50%), significantly outperforming MOGONET (mean AUC: 0.844–0.875, *p* < 0.001) and conventional machine learning models with tighter 95% confidence intervals, indicating superior stability despite the limited AD sample size. Clustering analysis revealed two distinct MCI subtypes with divergent molecular landscapes: Subtype A was enriched in energy metabolism and cellular maintenance pathways, whereas Subtype B was enriched in neuroinflammatory and aberrant signaling pathways. Notably, the majority of MCI patients who subsequently converted to AD were concentrated in the immune-inflammatory Subtype B. These findings highlight that neuroinflammation coupled with bioenergetic failure constitutes a critical mechanism driving the conversion from MCI to AD. Conclusions: The proposed methods not only provide the key multimodal biomarkers and enhance the accuracy of the classification model for early AD diagnosis but also identify biologically and clinically meaningful MCI subtypes with distinct molecular signatures and conversion risks. Exploring these associated multimodal biomarkers and MCI subtypes is of great significance, as they help elucidate the heterogeneous mechanisms underlying AD onset and progression, enable the identification of high-risk individuals likely to convert to AD, and provide a foundation for targeted therapeutic strategies and individualized clinical management. These findings have important implications for understanding disease heterogeneity, discovering potential intervention targets, and advancing precision medicine in neurodegenerative diseases.

## 1. Introduction

Alzheimer’s disease (AD), as a neurodegenerative disorder with insidious onset and difficulties in early detection, has a pathogenesis that remains incompletely understood, which severely limits the effectiveness of early diagnosis and intervention [1]. Imaging–transcriptomic analysis, which integrates multimodal neuroimaging with gene-expression data, offers a robust framework for investigating how molecular dysregulation is associated with large-scale brain structural and metabolic alterations in AD [2]. Advanced multimodal association methods, such as sparse canonical correlation analysis (SCCA) and its variants, enable the extraction of complementary imaging-transcriptomic features that serve as potential biomarkers for early AD diagnosis [2]. Nevertheless, these approaches differ in their emphasis on sparsity, adaptivity, or cross-modal association modeling, and each remains limited in representing nonlinear and heterogeneous relationships. Therefore, integrating multiple analytical strategies can yield a more comprehensive characterization of multimodal features, enhancing both the accuracy of early AD diagnosis and the understanding of underlying pathogenic mechanisms.

In the context of AD, imaging-transcriptomics not only enables the identification of deep associations between neuropathological changes and genetic variations but also provides new insights for discovering reliable early biomarkers [2,3]. However, despite substantial progress in multimodal feature association analyses, existing deep learning- and statistical-based imaging-transcriptomics approaches still face major challenges, including the effective handling of high-dimensional heterogeneous data, nonlinear feature representation across modalities, and the integration of prior biological knowledge [3]. These limitations hinder the accurate early diagnosis of AD and the comprehensive understanding of its heterogeneous progression.

Moreover, mild cognitive impairment (MCI), as the prodromal stage of AD, exhibits substantial clinical and molecular heterogeneity [4]. Investigating MCI subtypes is therefore crucial for elucidating distinct pathogenic pathways, identifying high-risk individuals who are more likely to progress to AD, and informing personalized prevention and therapeutic strategies [4]. The stratification of MCI based on multimodal imaging-transcriptomic features not only deepens our understanding of disease mechanisms but also supports precision medicine by enabling targeted interventions [4,5].

In recent years, multimodal association analysis methods, such as sparse canonical correlation analysis (SCCA), have been widely applied to identify associations between complex multivariate genetic and imaging features and to detect and predict AD-related biomarkers. For example, Du et al. proposed a variety of integration strategies with wide application scenarios based on different SCCA variations. Considering cross-omics association, they developed two multitask sparse canonical correlation analysis (inMTSCCA) methods for integrating multimodal data to identify genetic risk factors for AD by taking into account cross-omics association [6]. Considering linkage disequilibrium and computational efficiency, they proposed a multitask sparse canonical correlation analysis (MTSCCA) method for multimodal imaging-transcriptomics studies, which improved the ability to identify associations between genotypes and brain imaging phenotypes in multimodal data through group sparsity and joint individual feature selection, and validated its effectiveness in real data [7]. On the other hand, since the integration of prior information can improve model performance to some extent, Hao et al. proposed several models. For example, considering discriminative similarity information, they proposed a multimodal discriminative sparse canonical correlation analysis (MD-SCCA) algorithm, which associates discriminative similarity information to identify multimodal radiogenomic patterns associated with AD [8]. Despite these advances, Imaging-transcriptomics still faces challenges in high-dimensional data processing, nonlinear feature extraction, and integration of prior knowledge.

To address these challenges, we propose a two-stage framework that associates multimodal feature extraction with a graph-based classification model. In the first stage, we employ enhanced canonical correlation analysis (MFEAA) methods—including rAdaSMCCA and unAdaSMCCA—to associate sMRI, PET, and transcriptomic data, thereby identifying cross-modal associations and extracting the most discriminative multimodal biomarkers. In the second stage, these biomarkers are utilized in a graph convolutional network (GCN)-based classifier, further augmented with self-attention and self-expression mechanisms (GCNSASE), to improve diagnostic accuracy and capture complex intermodal relationships. Furthermore, an unsupervised clustering framework based on GCNSASE is adopted to uncover latent MCI subgroups characterized by distinct clinical and molecular features, and their differential risks of progression from MCI to AD are assessed. This innovative approach not only enables accurate classification between AD and MCI, but also allows for the biologically meaningful stratification of MCI patients into distinct subtypes, thereby offering new insights into the mechanisms underlying disease progression. In particular, our subtype analysis revealed two distinct MCI subtypes that differed significantly in both clinical outcomes and underlying biological pathways: one subtype was primarily associated with metabolic and developmental pathways and relatively stable cognition, whereas the other was characterized by immune- and stress-related responses and showed a higher risk of progression to AD. These findings highlight the heterogeneity within MCI and suggest that differential molecular mechanisms drive distinct disease trajectories, thereby enhancing risk prediction and providing a foundation for precision medicine strategies tailored to patient-specific disease mechanisms.

## 2. Methods

### 2.1. Overview of This Study

**Figure 1:**
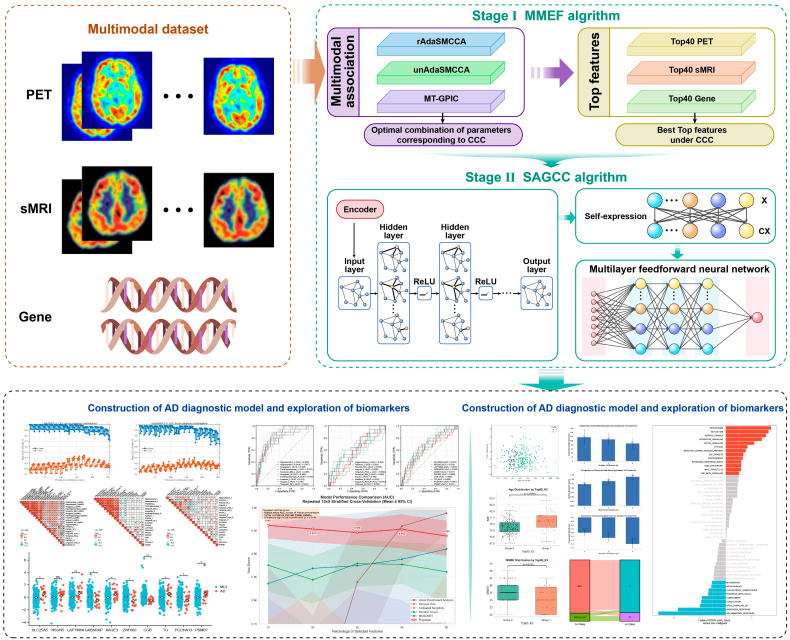
Schematic diagram of the overall algorithm process. * *p* < 0.05; ** *p* < 0.01; *** *p* < 0.001, ns, not significant.

The overview of the proposed two-stage model is shown in Figure 1. This model consists of two stages: in the first stage, improved multimodal feature extraction methods (such as rAdaSMCCA and unAdaSMCCA) are applied to associate PET, sMRI, and gene data and select optimal features; in the second stage, these features are fed into a neural network incorporating graph convolution, self-expression, and self-attention mechanisms, enabling accurate classification of AD and MCI, while unsupervised clustering further reveals potential MCI subtypes and their distinct risks of progression.

### 2.2. Stage I: Multimodal Feature Analysis Based on Association Algorithms (MFEAA)

#### 2.2.1. Three Improved Multimodal Correlation Feature Extraction Methods

*Algorithm principle of rAdaSMCCA*

To effectively associate multimodal data and identify key features associated with AD and MCI, this study employed a two-stage approach. In Stage I, we focused on multimodal feature fusion and extraction, aiming to capture the associations among sMRI, PET, and transcriptomic data. Canonical correlation analysis (CCA) was conducted on the ADNI database—sMRI, PET, and transcriptome data—to identify significant associations and evaluate the feature weights of each modality. Let $X_{1}\in R^{n\times p}$, $X_{2}\in R^{n\times q}\;and\;X_{3}\in R^{n\times r}$ represent the expression matrices for sMRI, PET, and transcriptome data, respectively. Here, *n* denotes the sample size, while *p*, *q*, and *r* represent the number of features for sMRI, PET, and genes, respectively.

Traditional methods such as sparse multi-view canonical correlation analysis (SMCCA) [9] often face challenges in handling the gradient dominance issue, which arises from the significant differences in correlation levels among multiple types of biomarkers. To address this challenge and improve the identification of multi-way associations among these heterogeneous biomarkers, an enhanced framework based on the robustness-aware adaptive sparse multi-view canonical correlation analysis (rAdaSMCCA) method [10] is applied in this study. Considering the nonlinearity, sparsity, and the need for adaptivity in handling the gradient dominance issue, this study incorporates a non-quadratic loss function into the SMCCA framework. The rAdaSMCCA method is designed to adaptively balance the contributions of different sub-objectives during the optimization process, thereby mitigating the impact of gradient dominance. The rAdaSMCCA method addresses the gradient dominance issue by employing a non-quadratic loss function, with the objective function given as follows:

(1) $$\underset{w_{1},w_{2},\ldots ,w_{K}}{min}\sum_{i<j}{∥X_{i}w_{i}-X_{j}w_{j}∥}_{2}+\sum_{k}\lambda_{k}\beta {∥w_{k}∥}_{FGL}+\lambda_{k}(1-\beta ){∥w_{k}∥}_{1}$$

The objective function of the proposed rAdaSMCCA method explicitly embodies both robustness-aware and adaptive characteristics. Specifically, the first term ${\left\|X_{i}w_{i}-X_{j}w_{j}\right\|}_{2}$ introduces a nonquadratic loss function, which mitigates the impact of extreme gradient dominance caused by large correlation discrepancies across modalities, thereby enhancing robustness against heterogeneous noise and outliers. To achieve adaptivity, the model employs modality-specific regularization parameters $\lambda_{k}$ and incorporates a weighted combination of the fused group Lasso penalty ${\left\|w_{k}\right\|}_{FGL}$ and the standard Lasso penalty ${\left\|w_{k}\right\|}_{1}$, controlled by the balancing parameter β. This design enables dynamic adjustment between joint and individual feature selection across different data views, allowing the model to flexibly adapt to varying data structures and sparsity patterns. Collectively, these components ensure that the optimization process is both robust to gradient dominance and adaptive to diverse biomarker characteristics.

*Algorithm principle of unAdaSMCCA*

Building on the foundation of addressing the gradient dominance issue in multi-way association analysis, this study further enhances the framework by introducing the uncertainty-aware adaptive sparse multi-view canonical correlation analysis (unAdaSMCCA) method [10]. This method goes beyond the rAdaSMCCA by not only employing a non-quadratic loss function but also explicitly considering the uncertainty associated with each sub-objective. By doing so, unAdaSMCCA assigns adaptive weights to different sub-objectives based on their estimation variances, thereby providing a more balanced and robust optimization strategy. The objective function of the unAdaSMCCA method is formulated as follows:

(2) $$\underset{w_{k}}{min}\sum_{1\leq i<j\leq 3}\frac{1}{2\sigma_{ij}^{2}}{∥X_{i}w_{i}-X_{j}w_{j}∥}_{2}^{2}+log\sigma_{ij}+\lambda_{1}\beta {∥w_{1}∥}_{FGL}+\lambda_{1}(1-\beta ){∥w_{1}∥}_{1}+\lambda_{2}∥w_{2}∥+\lambda_{3}∥w_{3}∥$$

Here, $\sigma_{ij}$ represents the estimated variance, which is used to measure the uncertainty of each sub-objective. The term $\frac{1}{2\sigma_{ij}^{2}}{∥X_{i}w_{i}-X_{j}w_{j}∥}_{2}^{2}$ is a weighted squared loss function, where the weights are determined by the inverse of the estimated variance. The term $log\sigma_{ij}$ serves as a regularization term for the estimated variance to prevent it from becoming excessively large.

#### 2.2.2. Multimodal Association and Feature Extraction

Within each cross-validation fold, gene features were first filtered by DEGs identified using limma on the fold-specific training partition only; this DEG filtering step was therefore fully nested inside the training-only cross-validation loop. In Stage I, multimodal association analysis was performed using two improved CCA-based algorithms, rAdaSMCCA and unAdaSMCCA, to identify cross-modal relationships among sMRI, PET, and transcriptome data. All analyses in this stage were conducted exclusively on the training set. To determine the optimal algorithm and hyperparameter configuration, a five-fold cross-validation procedure was applied within the training data. For each candidate algorithm and parameter combination, the mean CCC on the internal validation folds was computed and used as the evaluation metric. The configuration achieving the highest validation CCC was selected as the optimal model. Following model selection, the optimal CCA-based model was retrained on the entire training set to estimate canonical weights for each modality. Features were ranked according to the absolute values of their canonical weights, and the top-ranked multimodal features were selected as candidate biomarkers. These selected features, together with their modality-specific weights, were subsequently used as inputs for classification and subtyping analyses in Stage II.

Within each iteration of the repeated stratified 5-fold cross-validation, all preprocessing and model selection steps were performed using the training partition only. Specifically, Stage I feature ranking (including fold-specific DEG filtering) was fitted on the training partition and produced a ranked feature list; feature-ratio subsets (top 10–50%) were then selected from this training-derived ranking without accessing validation or test data. Stage II subsequently constructed the population graph using only the selected training features from the same partition; the adjacency matrix was computed and sparsified on training data only, and labels were never used in graph construction. Hyperparameters were tuned strictly within the development set under RSCV, and the held-out test set remained completely isolated until a single final confirmatory evaluation.

### 2.3. Stage II: Graph Convolutional Network with Self-Expression and Self-Attention (GCNSASE)

The proposed method introduces several notable innovations over conventional Graph Convolutional Networks (GCNs) and is designed to model complex inter-subject relationships using multimodal biomarkers. In this stage, a population-level graph is constructed, where each node represents one subject, and the node features correspond to the selected multimodal biomarkers (from sMRI, PET, or transcriptome data) extracted in Stage I. The graph is built globally within each training split and shared across subjects, rather than constructing an individual graph per subject.

Edges in the graph encode pairwise similarity between subjects based solely on their feature representations. Specifically, the adjacency matrix is computed using a feature-similarity measure (cosine similarity) applied to the node feature matrix within the training set. To ensure sparsity and computational efficiency, the similarity matrix is further sparsified using a k-nearest-neighbor (kNN) strategy, retaining only the top-k most similar neighbors for each node. Self-loops are added to preserve each node’s own information, and the resulting adjacency matrix is symmetrically normalized before being fed into the GCN. Importantly, class labels are never used in graph construction, and all graph-related parameters are estimated exclusively from the training data in each cross-validation fold, thereby avoiding information leakage from validation or test sets.

Given the constructed adjacency matrix, the GCN layer performs neighborhood aggregation to capture local connectivity patterns among subjects. The forward propagation of a graph convolution layer is defined as:

(3) H=ReLU(adj(XW)+b)

where W and b denote the trainable weight and bias parameters, and ReLU introduces nonlinearity. This step ensures that local connectivity patterns in the graph are preserved in the latent feature space.

To further enhance global representation learning, a self-expression layer is introduced after graph convolution. In this module, each node’s latent feature vector is reconstructed as a linear combination of latent features through a learned coefficient matrix:

(4) H=HC

where C is a coefficient matrix learned during training. Unlike classical subspace-clustering self-expression models that operate on the sample dimension, this formulation performs feature-wise self-expression, allowing the model to explicitly learn dependencies and interactions among latent feature dimensions. This mechanism strengthens global feature coupling and enriches the representation capacity of the network.

To adaptively emphasize informative features and subject relationships, a self-attention mechanism is incorporated. Query, key, and value projections are learned from the latent representations, and attention weights are computed via scaled dot-product interactions. The attention-based feature update is expressed as:

(5) H= attention_weights ×V

where V is the value matrix derived from the input features, and attention_weights are obtained through query-key interactions. This mechanism enables the model to dynamically focus on critical associations across modalities.

The proposed GCNSASE framework supports multi-view learning by initializing independent encoders and classifiers for each modality (sMRI, PET, and transcriptome data). Each view is optimized jointly using Adam optimizers, allowing complementary information from different modalities to be effectively integrated while preserving view-specific characteristics. The encoded features are subsequently passed to a fully connected classifier to output the probability distribution over diagnostic categories (AD vs. MCI). Table 1 presents the search range of the hyperparameters for GCNSASE.

### 2.4. Evaluation Protocol and Data Splitting Strategy

To address severe class imbalance and ensure statistical robustness, we implemented a hierarchical validation framework combining repeated stratified resampling with an independent held-out test set. Specifically, samples were first split into a development set (80%, n = 258) and a strictly held-out test set (20%, *n* = 65) using stratified sampling to preserve class distribution, with the latter reserved exclusively for final unbiased evaluation and completely isolated from all model selection, feature engineering, and hyperparameter tuning processes. All algorithm comparisons and biomarker selections were conducted within the development set using Repeated Stratified K-Fold Cross-Validation (10 repeats × 5 folds = 50 iterations), ensuring each fold maintained the original AD prevalence while aggregating predictions across 50 distinct validation sets to mitigate instability caused by the small AD sample size. Stage I feature ranking and Stage II classifier optimization were both evaluated via this RSCV framework, reporting mean AUC with standard deviation and bootstrapped 95% confidence intervals across all folds, and statistical significance between models was assessed using Wilcoxon signed-rank tests on paired CV results; only after finalizing architectures via RSCV was a single confirmatory evaluation performed on the held-out test set, ensuring that all “state-of-the-art” claims are substantiated by distributions derived from repeated resampling rather than single-split estimates.

## 3. Results

### 3.1. Dataset Acquisition and Preprocessing

The sMRI, PET and transcriptome data of AD and MCI used in this study were downloaded from the AD Neuroimaging Initiative (ADNI) website (https://adni.loni.usc.edu/), which included 323 non-Hispanic white participants with both transcriptomic data, PET, and sMRI. The dataset comprised 26 AD patients and 297 MCI patients. Among them, 272 MCI patients had complete clinical information, and 36 of them progressed to AD subsequently. It should be noted that the molecular modality used in this study corresponds to peripheral blood gene-expression profiles rather than genotype-level genetic variation; therefore, all subsequent multimodal association analyses reflect imaging-transcriptome relationships.

For sMRI data preprocessing, head motion correction was performed using DiffusionKit, and the corrected sMRI images were registered to the Montreal Neurological Institute (MNI) standard space. The segmentation was conducted using the Computational Anatomy Toolbox (CAT) in MATLAB software (Version R2023a, MathWorks, Natick, MA, USA, 2023), and 140 gray matter volumes of brain regions were extracted as imaging features based on the neuromorphometric atlas. For PET preprocessing, the MATLAB-based Statistical Parametric Mapping (SPM) toolbox was first used to co-register each participant’s FDG-PET image with their structural MRI image. We normalized the sMRI space to obtain the deformation field for each participant and applied it to the PET image to generate MNI-standardized PET images. Subsequently, we extracted gray matter from the FDG-PET brain tissue using the gray matter mask of the registered sMRI. The standardized uptake value ratio (SUVR) of 90 brain regions was used as the quantitative trait (QT) for FDG-PET imaging analysis.

Transcriptome data was obtained from peripheral blood samples collected using PAXgene tubes. Total RNA was extracted and hybridized to the Affymetrix Human Genome U219 Array platform. The raw expression data underwent rigorous quality control and preprocessing procedures. Specifically, raw intensity values were background-corrected, quantile-normalized, and summarized using the Robust Multi-array Average algorithm to generate gene-level expression values. To minimize technical variation, batch effects were corrected using the empirical Bayes method where applicable, ensuring comparability across samples processed at different time points. Probe sets were mapped to Human Genome Organization gene symbols using the “hgu219.db” package. Probes that did not map to a known gene or showed low expression levels across the majority of samples were filtered out to reduce noise. The final dataset represented gene-level mRNA expression profiles obtained from peripheral blood samples collected in PAXgene tubes. Total RNA was extracted and hybridized to the Affymetrix Human Genome U219 Array platform. Raw intensity data were background-corrected, quantile-normalized, and summarized using the Robust Multi-array Average algorithm. Batch effects were adjusted using an empirical Bayes method when necessary. Probe sets were annotated to Human Genome Organization gene symbols using the “hgu219.db” package, and unmapped or low-expressed probes were removed to reduce noise. To prevent label leakage, differential expression analysis (limma) and the resulting DEG-based gene filtering were performed exclusively within the training data. Specifically, within each inner cross-validation fold, limma was applied only to the fold-specific training partition (AD vs. MCI) to identify DEGs (*p* < 0.05 and |logFC| > 0.5), and only those DEGs were retained for subsequent Stage I association modeling in that fold. After model selection, limma was re-run once on the entire training split to generate the final DEG list used for training Stage I/II models; this yielded 779 DEGs under the same thresholds. The independent test split remained fully untouched and was never used for DEGs identification or any feature selection step.

### 3.2. Top Associated Features Identification of Stage I

**Figure 2:**
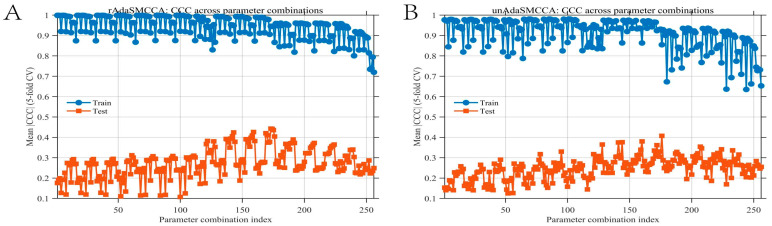
Parameter selection results for two CCA-based algorithms. (**A**) Five-fold cross-validation results of CCC for the rAdaSMCCA algorithm under different parameter combinations. The horizontal axis denotes the index of parameter combinations, and the vertical axis represents the average |CCC| obtained from five-fold cross-validation. Blue circles indicate results on the training set, while orange squares represent results on the internal validation set. (**B**) Five-fold cross-validation results of CCC for the unAdaSMCCA algorithm under different parameter combinations. The definitions of the horizontal and vertical axes are the same as in (**A**), with blue indicating training set performance and orange indicating internal validation set performance.

First, this study adopts a stratified sampling strategy to split the preprocessed sMRI, PET, and transcriptome data into a training set and an independent test set at an 8:2 ratio, ensuring that the class distributions (AD/MCI) remain consistent across the two sets. In the first stage, only the training data are used to evaluate two improved sparse multi-view canonical correlation analysis algorithms—rAdaSMCCA and unAdaSMCCA. To determine the optimal model and parameter configuration, five-fold cross-validation is conducted within the training set, with the canonical correlation coefficient (CCC) between the features and canonical variables used as the evaluation metric. Based on the cross-validation results, the optimal algorithm is selected, after which the canonical weights of features from each modality are computed. The test set was not accessed during Stage I model or feature selection. Features are then ranked according to the absolute values of their CCCs with the canonical variables, and the most discriminative Top-K feature subsets are selected. In the second stage, the key features identified in the first stage are used as inputs for extensive comparative experiments using Repeated Stratified Cross-Validation (RSCV) within the development set, and model performance is quantitatively evaluated using the area under the curve (AUC). Subsequently, two association analysis algorithms, rAdaSMCCA and unAdaSMCCA [10], were applied to train on the training set and evaluate the CCC on the internal validation partition. The algorithm parameter combinations ($\lambda_{1}$, $\lambda_{2}$ and $\lambda_{3}$) were selected from [0.01, 0.1, 1, 10], and Figure 2 illustrates the performance of different algorithms on both the training and internal validation sets. To evaluate the performance of different multimodal association analysis algorithms, we systematically compared rAdaSMCCA and unAdaSMCCA under various parameter combinations. Based on the parameter combination scan results shown in Figure 2, five-fold cross-validation was conducted for rAdaSMCCA and unAdaSMCCA on the training set and the internal validation set (via 5-fold cross-validation), with the mean CCC used as the criterion for model selection. Each point represents a distinct combination of regularization parameters, where blue indicates training performance and orange indicates internal validation performance. For rAdaSMCCA, under the parameter setting λ_1_ = 1, β = 1, λ_2_ = 10, and λ_3_ = 0.01, the model exhibited stable and relatively high correlations on the training set (mean CCC = 0.8965–0.9767, std = 0.0013–0.0079). The corresponding mean CCC values on the internal validation set ranged from 0.284 to 0.7161, with standard deviations between 0.0621 and 0.0905, indicating that this parameter combination maintains high training consistency while achieving relatively robust generalization performance on the internal validation data. For unAdaSMCCA, the optimal parameter combination was λ_1_ = 1, β = 1, λ_2_ = 10, and λ_3_ = 10. Under this configuration, the mean CCC on the training set ranged from 0.8295 to 0.8816 (std = 0.0156–0.0476), while the mean CCC on the internal validation set ranged from 0.1901 to 0.6847 (std = 0.0607–0.1386). Compared with rAdaSMCCA, unAdaSMCCA exhibited larger fluctuations in validation performance across different modalities, with notably higher uncertainty in the imaging modalities. Overall, rAdaSMCCA demonstrated more stable cross-fold consistency in the multimodal setting. The parameter combination yielding the highest validation CCC for rAdaSMCCA was therefore selected for subsequent feature extraction and association modeling.

Considering the space limitations, the top 10 extracted features for each modality are summarized in Table 2. Notably, the top 10 regions of interest (ROIs) for both sMRI and PET include the left and right hippocampus, which is consistent with existing findings. Specifically, significant atrophy of both the left and right hippocampus has been observed in patients with Alzheimer’s disease, representing one of the early pathological characteristics of the disorder. Compared with healthy individuals, patients with Alzheimer’s disease exhibit a marked reduction in hippocampal volume, with the left hippocampus typically showing more pronounced atrophy. In addition, the glucose metabolic rates of both the left and right hippocampus in Alzheimer’s disease patients are significantly lower than those in healthy controls. The hippocampus is a critical brain region responsible for memory and learning, and reduced glucose metabolism reflects impaired hippocampal function. Previous PET imaging studies have demonstrated decreased hippocampal glucose utilization in Alzheimer’s disease patients, which is closely associated with neuronal dysfunction and cognitive impairment.

For the Top ROIs identified from sMRI and PET, their anatomical distributions were visualized using the BrainNet Viewer (Version 1.7, Beijing Normal University, Beijing, N/A, China, 2016). The Top 10 ROIs derived from sMRI were primarily concentrated in the medial temporal lobe–limbic system and its adjacent cortices, including the bilateral hippocampus (Hippocampus_L/R), bilateral parahippocampal gyrus (ParaHippocampal_L/R), and bilateral amygdala (Amygdala_L/R). These regions further extended to the middle temporal gyrus (Temporal_Mid_L), inferior temporal gyrus (Temporal_Inf_L), fusiform gyrus (Fusiform_L), and angular gyrus (Angular_L), which are closely associated with memory retrieval, semantic processing, and visual integration. In contrast, the Top 10 ROIs identified from PET were more widely distributed across subcortical nuclei and the parietal–cingulate network, such as the pallidum (Pallidum_L/R), putamen (Putamen_R), inferior parietal lobule (Parietal_Inf_L), angular gyrus (Angular_L), posterior cingulate gyrus (Cingulum_Post_L/R), and insula (Insula_L). These findings suggest that metabolic abnormalities are not limited to memory-related medial temporal lobe structures but may also involve more extensive cortical–subcortical circuits.

**Figure 3:**
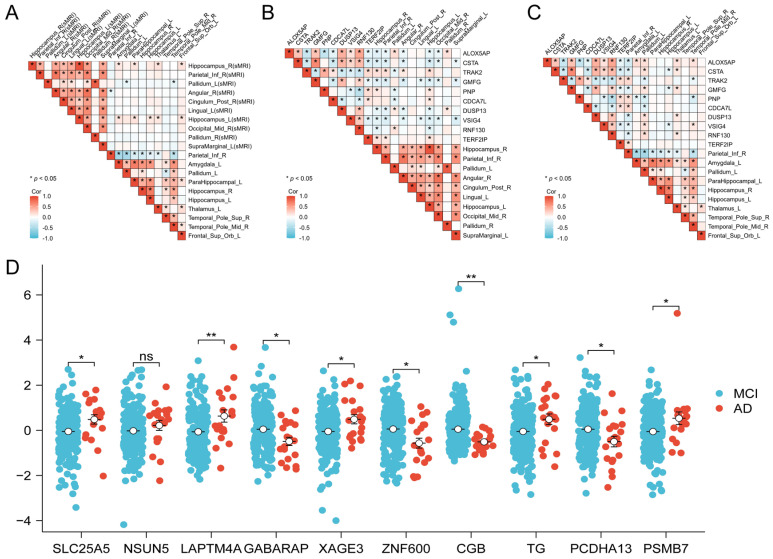
Correlation analysis of top markers. (**A**–**C**) Heatmaps of correlations between sMRI–PET, sMRI–gene, and PET–gene. (**D**) Boxplot showing the differences-in-differences of the top 10 genes between the MCI and AD groups. * *p* < 0.05; ** *p* < 0.01; ns, not significant.

To investigate cross-modal associations, pairwise correlation analyses were further conducted among the three data modalities, and representative cross-modal pairings are presented in Figure 3A–C. The feature selection results show that the hippocampus (Hippocampus_L) consistently ranks among the top features in both sMRI and PET modalities, suggesting that this region may exhibit synchronized structural alterations and metabolic changes. In addition, limbic system features identified from sMRI, represented by the amygdala and parahippocampal gyrus, co-occur within the same feature set as key nodes of the default mode network and salience network identified from PET, such as the insula, posterior cingulate gyrus, and inferior parietal lobule/angular gyrus. These observations imply the presence of tightly coupled cross-modal alterations between emotion–memory-related circuits and higher-order cognitive networks.

In the genetic modality (Figure 3D), the top 10 genes span multiple pathways that may be functionally linked to brain phenotypes. For example, SLC25A5, a gene involved in mitochondrial adenine nucleotide transport, may be associated with energy metabolism and neuronal vulnerability. GABARAP is related to autophagy and vesicle trafficking processes, potentially reflecting alterations in protein homeostasis and intracellular clearance mechanisms. PSMB7, a component of the proteasome system, suggests the potential involvement of protein degradation pathways. In addition, PCDHA13, a member of the protocadherin family, may be implicated in synaptic connectivity and neural circuit plasticity. Collectively, the selected multimodal features not only exhibit strong discriminative or explanatory power within their respective modalities but also provide candidate clues for elucidating the potential biological links between imaging phenotypes and molecular-level mechanisms.

### 3.3. Classification Results of Stage II

In Stage II, the top-ranked multimodal features (sMRI, PET, and transcriptome data) extracted from Stage I were utilized to construct diagnostic classifiers for distinguishing AD from MCI. Given the significant class imbalance in the dataset, we implemented a rigorous repeated stratified cross-validation (RSCV) scheme to ensure statistical robustness and generalizability. Specifically, we employed a 5-fold stratified cross-validation repeated 10 times (50 total evaluations), ensuring that each fold maintained the original class distribution. This approach addresses the instability concerns arising from the small number of AD cases by providing a distribution of performance estimates rather than a single point estimate.

**Figure 4:**
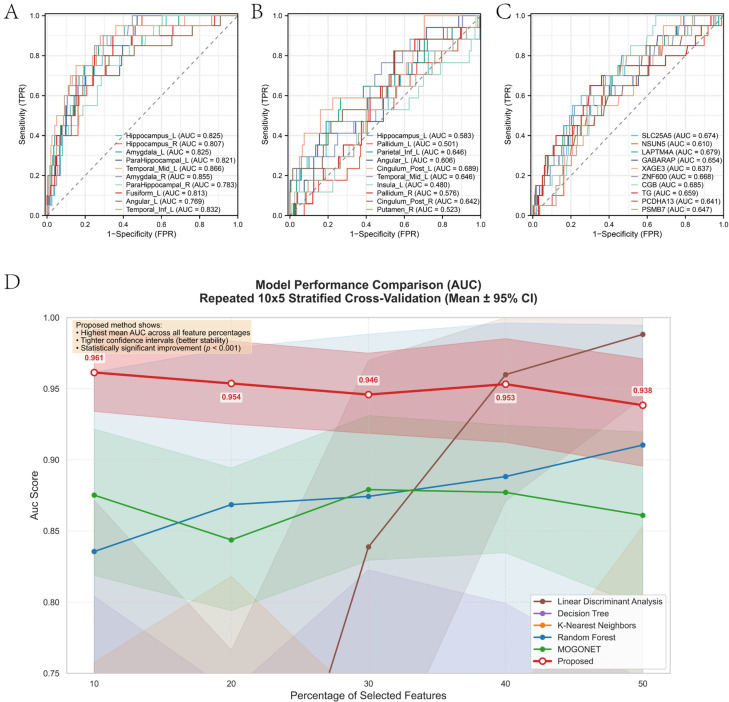
Comparison of classification performance between the proposed method and baseline algorithms. (**A**–**C**) Representative ROC curves for individual modalities. The dashed diagonal line represents the line of no discrimination (random classifier, AUC = 0.5). (**D**) Mean AUC (solid lines) with 95% confidence intervals (shaded areas) across repeated stratified cross-validation (10 repeats × 5 folds) as a function of feature percentage. The proposed method demonstrates consistently superior performance and tighter confidence intervals, indicating both higher accuracy and greater stability.

Feature subsets ranging from the top 10% to 50% (ranked by mutual information in Stage I) were selected from each modality to systematically evaluate classification performance. These features were input into our proposed Graph Convolutional Classifier with Self-Attention and Self-Expression. For comprehensive benchmarking, we compared our method against conventional machine learning algorithms (Random Forest, K-Nearest Neighbors, Decision Tree, Linear Discriminant Analysis) and the state-of-the-art deep learning method MOGONET, all evaluated under identical RSCV conditions. Performance was quantified using AUC with bootstrapped 95% confidence intervals (CIs) and standard deviation (SD) across the 50 CV folds. As summarized in Figure 4 and Table 3, the proposed method achieved state-of-the-art performance across all feature proportions, with mean AUC values ranging from 0.938 to 0.961, substantially outperforming MOGONET (mean AUC: 0.844–0.879) and conventional classifiers.

Notably, statistical significance was assessed using the Wilcoxon signed-rank test on paired CV fold results. At the 50% feature level, the proposed method significantly outperformed MOGONET (mean difference: +0.077, *p* = 1.78 × 10^−15^) and showed marginally significant improvement over Random Forest (mean difference: +0.028, *p* = 0.084). The proposed method also exhibited superior stability, with consistently lower standard deviations (SD: 0.015–0.021) compared to MOGONET (SD: 0.025–0.032) and Random Forest (SD: 0.080–0.090), indicating robust performance across different data splits despite the extreme class imbalance. Interestingly, while Linear Discriminant Analysis (LDA) showed high AUC values at 40–50% features (0.960–0.988), its performance was highly unstable at lower feature dimensions (AUC dropped to 0.529 at 20%), suggesting potential overfitting to high-dimensional inputs. In contrast, the proposed method maintained stable, high performance across all feature subsets.

### 3.4. Subtyping of MCI Based on Multimodal Top Biomarkers

**Figure 5:**
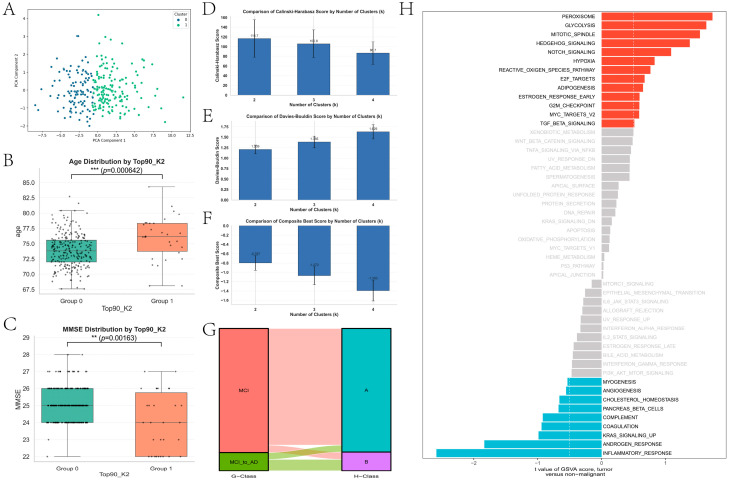
Subtype identification results of AD and MCI. (**A**) Subtype identification of AD and MCI samples based on the top 90 features. (**B**,**C**) Boxplots of MMSE and age distributions between the two subtypes. (**D**–**F**) CH index, DB index, and silhouette score for different cluster numbers (k = 2–4). (**G**) Sankey diagram showing the distribution of non-converting MCI and MCI-to-AD samples across different subtypes. Colors represent different subgroups and their transitions. Red/pink and cyan/purple denote distinct classes, and the connecting bands indicate the proportion of samples between groups. (**H**) GSVA analysis results of pathway enrichment differences between the two subtypes. ** *p* < 0.01; *** *p* < 0.001; ns, not significant. Red bars indicate positively enriched pathways, blue bars indicate negatively enriched pathways, and gray bars indicate non-significant pathways.

Although the classification results in the second stage indicate that the model can effectively distinguish between AD and MCI, the heterogeneity within the MCI population still requires further characterization. Not all individuals with MCI progress to AD; therefore, identifying subgroups with distinct clinical phenotypes and molecular mechanisms is crucial for disease stratification and risk prediction. Based on the multimodal features of MCI samples obtained in the first stage, unsupervised clustering was performed on the Top 90 features integrated across the three modalities, and internal clustering metrics were compared for different numbers of clusters k. The results showed that k = 2 achieved a higher Calinski–Harabasz score, a lower Davies–Bouldin score, and the best overall performance. Accordingly, a two-cluster solution was selected as the optimal scheme. PCA-based visualization further demonstrated clear separability between the two clusters in a low-dimensional space (Figure 5A). The two MCI subtypes were therefore denoted as subtype A and subtype B. Significant differences in clinical characteristics were observed between the two subtypes. Subtype B was associated with older age (Figure 5B, *p* = 0.000642) and lower MMSE scores (Figure 5C, *p* = 0.00163), indicating more severe cognitive impairment and a clinical profile closer to an unfavorable disease trajectory. Further pathway-level analyses revealed distinct molecular mechanisms underlying the two subtypes. Subtype A showed greater enrichment in metabolism-, proliferation-, and development-related pathways, such as Peroxisome, Glycolysis, and Notch/Hedgehog signaling, whereas subtype B was characterized by stronger enrichment of immune- and inflammation-related processes, including inflammatory response, complement/coagulation, interferon response, IL6/JAK/STAT3 signaling, and angiogenesis (Figure 5H). Regarding the distribution of MCI-to-AD conversion, the Sankey diagram indicated that individuals who converted from MCI to AD were more likely to be classified into subtype B (Figure 5G), suggesting that this subtype is associated with a higher risk of progression to AD.

### 3.5. Ablation Study and Component Analysis

To further evaluate the contribution of each component in the proposed framework, ablation experiments were conducted by systematically removing or modifying specific modules (Table 4). Crucially, to ensure fair comparison and statistical rigor, all ablation variants were evaluated using the same Repeated Stratified Cross-Validation (RSCV) protocol (10 repeats × 5 folds) as the main benchmarks. The complete model (Case 0), which integrates multimodal feature extraction, graph convolution, self-attention, and a self-expression layer, achieved the best performance with a mean AUC of 0.955 ± 0.015 [0.916, 0.974]. When the CCA-based feature extraction module was removed (Case 1), the mean AUC dropped sharply to 0.634 ± 0.008 [0.584, 0.701], highlighting the critical role of feature extraction in enhancing cross-modal associations. Excluding the attention mechanism (Case 2) also resulted in performance degradation, with a mean 0.690 ± 0.011 [0.617, 0.753], demonstrating the importance of attention in emphasizing key cross-modal relationships. In addition, experiments that varied the hidden dimensionality of the classifier showed that both 512 dimensions (Case 3: 0.705 ± 0.034 [0.649, 0.773]) and 1024 dimensions (Case 4: 0.699 ± 0.021 [0.628, 0.764]) led to inferior performance compared with the complete model, indicating that excessively high dimensionality introduces redundancy and impairs generalization ability.

## 4. Discussion

In this study, we identified reliable biomarkers for AD and constructed a two-stage diagnostic model by integrating multimodal data with advanced machine learning algorithms. Beyond diagnostic accuracy, our framework also enabled the stratification of MCI patients into distinct subtypes with differing clinical characteristics and risks of progression to AD. These results highlight the heterogeneity within MCI and provide new insights into the biological mechanisms underlying disease progression, underscoring the value of subtype-specific approaches for early diagnosis and precision medicine.

First, the two-stage algorithm proposed in this study successfully integrated multimodal neuroimaging and transcriptome data, identifying biomarkers that maximize the concordance correlation coefficient. In terms of neuroimaging, our method detected Hippocampus_L, Angular_L, and Temporal_Mid_L as common critical regions across sMRI and PET modalities. The hippocampus is central to memory formation, and its atrophy is a hallmark of AD [11,12], while the angular gyrus, a key node in the Default Mode Network, has been shown to exhibit functional connectivity disruptions in AD [13] and is a target for therapeutic rTMS [14]. Crucially, the top extracted genes illuminate specific molecular deficits in AD pathology: SLC25A5 (encoding ANT2) is implicated in mitochondrial ADP/ATP exchange, reflecting the mitochondrial dysfunction and energy hypometabolism characteristic of AD neurons [15]; GABARAP mediates autophagy and receptor trafficking, processes essential for the clearance of amyloid-beta aggregates and maintenance of synaptic balance [16]; and PSMB7, a proteasome subunit, points to the failure of the ubiquitin-proteasome system to degrade toxic protein aggregates, a core pathological mechanism in neurodegeneration [17]. Collectively, these biomarkers not only demonstrated excellent classification performance via the GCNSASE model but also provided biologically interpretable insights into the structural, metabolic, and molecular heterogeneity of Alzheimer’s disease.

However, in clinical practice, merely distinguishing AD from MCI is insufficient to meet the demands of precision medicine. As the prodromal stage of AD, MCI offers greater potential for intervention, yet its progression is highly heterogeneous: some patients convert to AD within a few years, whereas others remain stable over extended periods [18]. Therefore, beyond the binary classification of AD and MCI, further exploration of potential MCI subtypes is critical for identifying high-risk individuals and elucidating distinct mechanisms of disease progression [19]. In this study, we employed unsupervised clustering to characterize MCI subtypes and identified two groups with significant differences in clinical features and conversion risk. GSVA analysis revealed distinct biological landscapes between the two subtypes. Subtype B was primarily enriched in immune inflammation and signaling pathways, exhibiting significant upregulation of Inflammatory Response, IL6-JAK-STAT3 signaling, and PI3K-AKT-mTOR signaling. In contrast, subtype A was enriched in pathways that maintain cellular function and energy metabolism, most notably Oxidative Phosphorylation, DNA Repair, and Myc Targets, all of which were significantly downregulated in subtype B. Notably, MCI patients who later progressed to AD were primarily concentrated in subtype B. The activation of the IL6-JAK-STAT3 pathway in this subtype suggests the presence of a chronic neuroinflammatory state, which activates microglia and astrocytes, thereby exacerbating synaptic loss and neuronal death [20,21]. Additionally, the enrichment of PI3K-AKT-mTOR signaling in the high-risk group aligns with recent findings that abnormal mTOR activation inhibits autophagy, leading to the accumulation of β-amyloid (Aβ) and tau oligomers [22,23]. In contrast, the suppression of Oxidative Phosphorylation in subtype B (or its preservation in subtype A) points to mitochondrial dysfunction—an early event in AD pathology, with bioenergetic failure occurring prior to clinical symptoms [24]. Therefore, subtype B represents a “bioenergetic dysfunction with inflammation” phenotype, highlighting the potential of targeting neuroinflammation and metabolic regulators in delaying the conversion from MCI to AD.

Despite these promising results, several limitations should be acknowledged. First, the sample size, particularly the number of AD patients, was relatively small, which may limit the generalizability of the clustering outcomes. Second, although the model associated multimodal data, the present analysis was restricted to sMRI, PET, and transcriptome data, without incorporating other potentially informative modalities such as proteomics, metabolomics, or longitudinal follow-up data. Third, while the clustering results demonstrated biological interpretability, they require validation in independent cohorts and linkage with longitudinal clinical outcomes. Future studies should focus on expanding the sample size to include larger and more diverse populations, integrating additional omics layers and fluid biomarkers, and experimentally validating the subtype-specific pathways. Moreover, incorporating longitudinal trajectory analyses of MCI patients will further improve the prediction of conversion risk and refine subtype definitions. Ultimately, combining robust classification with biologically meaningful subtype identification holds promise for advancing precision medicine in AD, enabling earlier diagnosis, more individualized intervention, and optimized clinical trial design.

## 5. Conclusions

This study proposed a two-stage multimodal analytical framework, MFEAA-GCNSASE, that integrates multimodal imaging-transcriptome analysis and machine learning to address the challenges of AD, including its complex etiology, high heterogeneity, and difficulty in early diagnosis. In the first stage, multiple imaging–genetic association analysis methods (MFEAA), including rAdaSMCCA and unAdaSMCCA, were integrated to extract complementary multimodal features from sMRI, PET, and transcriptome data, thereby identifying biologically interpretable biomarkers reflecting structural, functional, and molecular associations. In the second stage, a GCC incorporating self-attention and self-expression mechanisms (GCNSASE) was constructed to enhance multimodal feature representation and improve diagnostic accuracy. Experimental results demonstrated that the proposed framework achieved superior performance in classifying AD and MCI and revealed two biologically and clinically distinct MCI subtypes through unsupervised clustering based on the top biomarkers. Subtype A was enriched in energy metabolism and cellular maintenance pathways, specifically Oxidative Phosphorylation and DNA Repair, and exhibited relatively stable cognitive performance, whereas Subtype B was enriched in neuroinflammatory and aberrant signaling pathways, notably IL6-JAK-STAT3 and PI3K-AKT-mTOR signaling, and included most patients who subsequently converted to AD. These findings suggest that immune dysregulation coupled with bioenergetic failure may be the key mechanisms driving the progression of AD. In summary, the proposed multimodal integrative framework demonstrates significant potential for early AD diagnosis, MCI subtype identification, and elucidation of disease mechanisms, providing new insights and methodological support for individualized risk prediction and precision intervention in neurodegenerative disorders.

## Figures and Tables

**Table 1:** The hyperparameter search of GCNSASE.

Component	Hyperparameter	Value/Search Space
Graph construction	Similarity metric	cosine
Graph construction	Sparsification	kNN: k ∈ {5, 10, 15, 20} or threshold τ ∈ {…}
Graph construction	Self-loops	A ← A + I
Graph construction	Normalization	(D^−1/2^)
Feature selection	Feature ratio r	r ∈ {10%, 20%, 30%, 40%, 50%}
Encoder (Proposed)	hidden_dim	{64, 128, 256, 512}
Optimizer	Adam learning rate (encoder)	{1 × 10^−5^, 1 × 10^−4^, 5 × 10^−4^, 1 × 10^−3^}
Optimizer	Adam learning rate (classifier)	{1 × 10^−5^, 1 × 10^−4^, 5 × 10^−4^, 1 × 10^−3^}
Dropout (baseline)	gcn_dropout	{0.0, 0.3, 0.5, 0.7}
Activation	LeakyReLU slope	0.25
Training	Epochs	200
Training	Weight decay	{0, 1 × 10^−5^, 1 × 10^−4^, 1 × 10^−3^}
Randomness	Seeds	{0,…,9}

**Table 2:** Top 10 extracted features of each modality.

Top 10 ROI (sMRI)	Top 10 ROI (PET)	Top 10 Genes
Hippocampus_L	Hippocampus_L	SLC25A5
Hippocampus_R	Pallidum_L	NSUN5
Amygdala_L	Parietal_Inf_L	LAPTM4A
ParaHippocampal_L	Angular_L	GABARAP
Temporal_Mid_L	Cingulum_Post_L	XAGE3
Amygdala_R	Temporal_Mid_L	ZNF600
ParaHippocampal_R	Insula_L	CGB
Fusiform_L	Pallidum_R	TG
Angular_L	Cingulum_Post_R	PCDHA13
Temporal_Inf_L	Putamen_R	PSMB7

**Table 3:** Classification performance summary (Mean AUC ± SD [95% CI]) based on repeated stratified 5-fold cross-validation (n = 50 iterations).

Model	10% Features	20% Features	30% Features	40% Features	50% Features
Proposed	0.961 ± 0.015 [0.934, 0.992]	0.954 ± 0.017 [0.925, 0.984]	0.946 ± 0.016 [0.919, 0.975]	0.953 ± 0.021 [0.912, 0.985]	0.938 ± 0.020 [0.896, 0.971]
MOGONET	0.875 ± 0.028 [0.819, 0.922]	0.844 ± 0.028 [0.794, 0.894]	0.879 ± 0.032 [0.829, 0.931]	0.877 ± 0.025 [0.835, 0.924]	0.861 ± 0.031 [0.797, 0.920]
Random Forest	0.835 ± 0.090 [0.648, 0.962]	0.868 ± 0.083 [0.681, 0.979]	0.874 ± 0.090 [0.675, 0.988]	0.888 ± 0.084 [0.728, 0.996]	0.910 ± 0.080 [0.742, 0.995]
Linear Discriminant Analysis	0.685 ± 0.128 [0.421, 0.871]	0.529 ± 0.128 [0.297, 0.766]	0.839 ± 0.090 [0.659, 0.970]	0.960 ± 0.044 [0.871, 1.000]	0.988 ± 0.017 [0.944, 1.000]
K-Nearest Neighbors	0.547 ± 0.098 [0.384, 0.758]	0.631 ± 0.114 [0.388, 0.818]	0.575 ± 0.103 [0.387, 0.725]	0.588 ± 0.092 [0.418, 0.725]	0.662 ± 0.104 [0.456, 0.853]
Decision Tree	0.548 ± 0.100 [0.447, 0.804]	0.566 ± 0.080 [0.452, 0.737]	0.580 ± 0.104 [0.447, 0.823]	0.581 ± 0.096 [0.452, 0.799]	0.581 ± 0.091 [0.449, 0.745]

**Table 4:** Results of the ablation experiment (Mean AUC ± SD across 50 RSCV folds).

Case	Mean AUC ± SD [95% CI]
Case 0	0.955 ± 0.015 [0.916, 0.974]
Case 1	0.634 ± 0.008 [0.584, 0.701]
Case 2	0.690 ± 0.011 [0.617, 0.753]
Case 3	0.705 ± 0.034 [0.649, 0.773]
Case 4	0.699 ± 0.021 [0.628, 0.764]

## Data Availability

The authors gratefully acknowledge the Alzheimer’s Disease Neuroimaging Initiative (ADNI) for providing the multimodal imaging and genetic data used in this research (https://adni.loni.usc.edu). The dataset consisted of 323 participants with available sMRI, PET, and transcriptome data. All data were accessed and processed following ADNI data use and publication guidelines. The model codes used in this study are publicly available at: https://jihulab.com/group133902/project.
